# PSMA3-AS1 induced by transcription factor PAX5 promotes cholangiocarcinoma proliferation, migration and invasion by sponging miR-376a-3p to up-regulate LAMC1

**DOI:** 10.18632/aging.203828

**Published:** 2022-01-12

**Authors:** Dongsheng Sun, Fujun Li, Lang Liu, Shaobo Yu, Haicun Wang, Xin Gao, Guanglin Liu, Yuqiao Zhao, Gongcai Qiu, Xingming Jiang

**Affiliations:** 1 Department of General Surgery, The Second Affiliated Hospital of Harbin Medical University, Harbin 150086, China; 2 Department of General Surgery, South China Hospital, Health Science Center, Shenzhen University, Shenzhen 518116, China

**Keywords:** PSMA3-AS1, cholangiocarcinoma, lncRNA, miR-376a-3p, LAMC1

## Abstract

Long noncoding RNAs (lncRNAs) have been reported to exhibit a crucial regulatory role in tumor progression, including cholangiocarcinoma (CCA). As a promising lncRNA, proteasome 20S subunit alpha 3 antisense RNA 1 (PSMA3-AS1) is involved in development of various tumors. However, the role and function of PSMA3-AS1 in CCA remain unclear. The aim of this study is to examine the expression, function, mechanism, and clinical significance of PSMA3-AS1 in CCA development. By TCGA database analysis, we found that PSMA3-AS1 was overexpressed in CCA. Consistent with the TCGA analysis, PSMA3-AS1 was significantly overexpressed in CCA tissues and cells by RT-qPCR. Upregulated PSMA3-AS1 was related to lymph node invasion, advanced TNM stage and poor survival, and was an independent risk factor of prognosis for CCA patients. Functionally, CCK-8, EdU and colony formation assays confirmed that upregulated PSMA3-AS1 promoted CCA cell proliferation, whereas downregulated PSMA3-AS1 inhibited proliferation. This result was further confirmed by subcutaneous tumor formation in nude mice. Wound healing and transwell assays confirmed that increased PSMA3-AS1 promoted CCA cell migration and invasion, whereas decreased PSMA3-AS1 inhibited these biological phenotypes. In addition, PSMA3-AS1 promoted the EMT process of CCA by downregulating E-cadherin and upregulating N-cadherin and vimentin. Mechanistically, transcription factor PAX5 bound to the promoter region of PSMA3-AS1 and promoted its transcription. Simultaneously, PSMA3-AS1 primarily localized in the cytoplasm could competitively bind miR-376a-3p to upregulate LAMC1, thereby accelerating CCA progression. This study uncovers that PSMA3-AS1 functions as a cancer-promoting gene in CCA, and PAX5/PSMA3-AS1/miR-376a-3p/LAMC1 axis plays a vital role in CCA development.

## INTRODUCTION

Cholangiocarcinoma (CCA) is a malignant tumor that happens in the biliary tree. In recent years, the incidence of CCA is gradually increased, especially in Thailand [1]. CCA has a high malignant degree and an insidious incidence. Due to the lack of sensitive and specific early diagnostic markers, most patients were already the later period of the carcinoma [2]. Thus they lost the opportunity for radical surgery. The long-term survival and prognosis of patients with CCA were quite poor [3]. Accordingly, exploring new tumor markers is urgently needed, and finding new anticancer targets for CCA patients with advanced stage is also a practical problem that needs to be solved clinically.

Long noncoding RNA (lncRNA) is a linear RNA molecule longer than 200 nt [4]. They are not able to encode proteins owing to absence of full ORF [5]. LncRNAs are involved in gene expression regulation in the form of RNA and particularly exert crucial action in tumorigenesis, including CCA [6, 7]. For instance, lncRNA HOXD-AS1 promoted CCA cell growth and metastasis through adsorbing miR-520c-3p [4]; lncRNA MEG3 was downexpressed and suppressed cellular malignant biological behaviors in CCA [8]. Proteasome 20S subunit alpha 3 antisense RNA 1 (PSMA3-AS1) is a tumor-related lncRNA located on chromosome 14q23.1 [9]. PSMA3-AS1 is an oncogene and exerts pro-cancer function in colorectal cancer, esophageal cancer and glioma [10–12]. For example, PSMA3-AS1 boosted metastasis of colorectal cancer cell by restraining miR-4429 [10]; PSMA3-AS1 adsorbed miR-101 to promote esophageal tumor development [11]. Nevertheless, function and mechanism of PSMA3-AS1 in CCA are still obscure.

The paired box 5 (PAX5) belongs to paired box domain family, which is mapping to 9p13 [13]. The aberrant expression of PAX5 is associated with multiple carcinomas [14]. For example, PAX5 as transcription factor induced the overexpression of IDH1-AS1 to promote prostate tumor proliferation [15]. The laminin subunit gamma 1 (LAMC1) is a member of laminin family. It is involved in basement membrane reestablishment and cancer invasion [16]. For instance, LAMC1 boosted tumor cell proliferation and inhibited cell death in hepatocellular carcinoma [17]. The correlation of PSMA3-AS1, PAX5 and LAMC1, as well as their function in CCA is not reported and requires further investigation.

The present study confirmed a significantly high expression of PSMA3-AS1 in CCA. Elevating PSMA3-AS1 was linked to clinicopathological characteristics and worse survival. Furthermore, PSMA3-AS1 competitively adsorbed miR-376a-3p to upregulate LAMC1, thereby promoting CCA cell growth and metastasis. In addition, PSMA3-AS1 was transcriptionally activated by PAX5. These findings suggest that PSMA3-AS1 is expected to become viable intervention target in CCA.

## RESULTS

### PSMA3-AS1 expression in CCA and the association with survival

**Figure 1:**
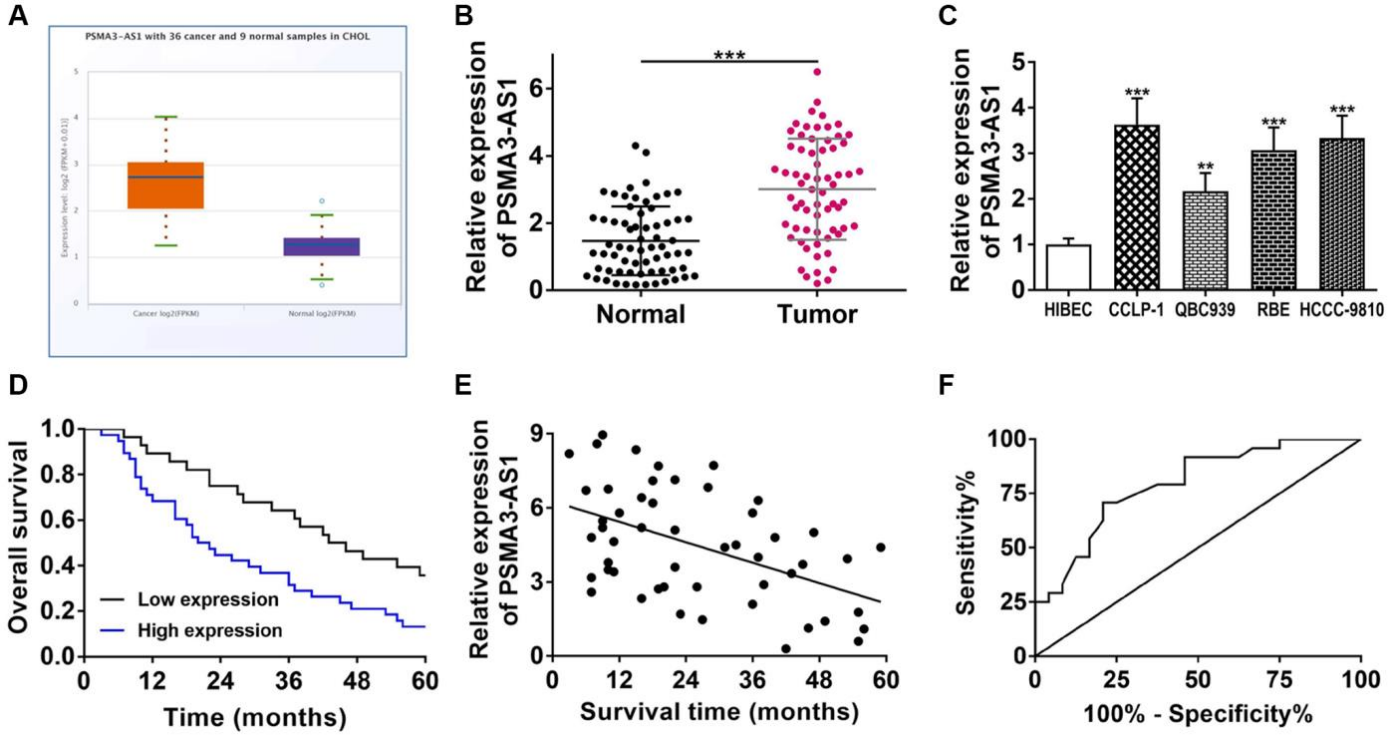
**PSMA3-AS1 expression and its association with clinicopathological characteristics.** (**A**) PSMA3-AS1 expression in TCGA database. (**B**) PSMA3-AS1 expression in CCA tissues and paired adjacent tumor-free bile duct tissues. (**C**) PSMA3-AS1 expression in CCA cells. (**D**) Kaplan-Meier curves analyzed the survival correlation. (**E**) Pearson correlation analysis examined the linear correlation of PSMA3-AS1 expression and survival time. (**F**) ROC curve assessed the potential of PSMA3-AS1 as a prognostic marker. ^**^P < 0.01, ^***^P < 0.001.

Via TCGA database analysis, we found that PSMA3-AS1 was highly expressed in cholangiocarcinoma (*P* < 0.001, Figure 1A). Therefore, we decided to further investigate its role in CCA. By RT-qPCR detection, PSMA3-AS1 was indeed highly expressed in cholangiocarcinoma tissues and cells (Figure 1B, 1C). In the light of average value of PSMA3-AS1 expression, we divided 66 CCA patients into two sets. Clinicopathological correlation analysis showed that highly expressed PSMA3-AS1 was linked to advanced TNM stage and lymph node invasion of CCA patients (Table 1). Kaplan-Meier survival curve displayed that elevating PSMA3-AS1 patients possessed shorter survival time (log rank *P* < 0.001, Figure 1D). Pearson correlation analysis further confirmed a negative correlation of PSMA3-AS1 expression and survival time (r = −0.4915, *P* < 0.001, Figure 1E). The univariate COX regression analysis showed that PSMA3-AS1, lymph node invasion, and advanced TNM stage were related to overall survival, and multivariate analysis further revealed that PSMA3-AS1 expression as well as advanced TNM stage was an independent risk factor for prognosis (Table 2). The ROC curve revealed that AUC value of PSMA3-AS1 as prognostic marker was 0.793 (95% CI: 0.668–0.919) with 70.8% sensitivity and 79.2% specificity (*P* < 0.001, Figure 1F). These results suggest that highly expressed PSMA3-AS1 is significantly linked to survival, and can serve as an effective indicator for prognostic evaluation. In addition, the interference efficiency of si-PSMA3-AS1 and oe-PSMA3-AS1 were detected for next cytology experiments (Supplementary Figure 1A).

**Table 1:** Correlation between PSMA3-AS1 expression and clinicopathological characteristics of CCA patients.

**Clinicopathological parameters**	**Total**	**PSMA3-AS1 expression**		***P*-value**
		**Low**	**High**	
Age (years)				
<60	19	11	8	0.106
≥60	47	17	30
Gender				
Male	29	14	15	0.394
Female	37	14	23
Differentiation grade				
Well/moderate	28	15	13	0.116
Poor/undifferentiated	38	13	25
Serum CA19-9 level				
≤37 U/ml	25	13	12	0.219
>37 U/ml	41	15	26
Serum CEA level				
≤5 ng/ml	23	13	10	0.090
>5 ng/ml	43	15	28
TNM stage				
I–II	20	13	7	0.014^*^
III–IV	46	15	31
Lymph node invasion				
No	27	16	11	0.021^*^
Yes	39	12	27

^*^P < 0.05. Abbreviations: PSMA3-AS1: proteasome subunit α3 antisense RNA 1; CCA: cholangiocarcinoma; CA19-9: carbohydrate antigen 19-9; CEA: carcinoembryonic antigen.

**Table 2:** Univariate and multivariate analysis for overall survival of CCA patients.

**Variables**	**Univariate analysis**			**Multivariate analysis**		
	**HR**	**95% CI**	***P*-value**	**HR**	**95% CI**	***P*-value**
Age (years) ≥60 vs. <60	1.579	0.900–2.768	0.111			
Gender Male vs. Female	1.331	0.753–2.354	0.325			
Differentiation grade Poor/undifferentiated vs. Well/moderate	1.436	0.840–2.453	0.186			
Serum CA19-9 level >37 U/ml vs. ≤37 U/ml	1.364	0.764–2.434	0.294			
Serum CEA level >5 ng/ml vs. ≤5 ng/ml	1.517	0.879–2.620	0.135			
TNM stage III–IV vs. I–II	1.893	1.100–3.258	0.021^*^	2.145	1.182–3.892	0.012^*^
Lymph node invasion Yes vs. No	1.852	1.045–3.282	0.035^*^	1.686	0.969–2.935	0.065
PSMA3-AS1 expression Low vs. High	2.059	1.159–3.658	0.014^*^	2.195	1.246–3.867	0.006^**^

^*^P < 0.05, ^**^P < 0.01. Abbreviations: CCA: cholangiocarcinoma; HR: hazard ratio; CI: confidence interval; CA19-9: carbohydrate antigen 19-9; CEA: carcinoembryonic antigen; PSMA3-AS1: proteasome subunit α3 antisense RNA 1.

### PSMA3-AS1 boosted cholangiocarcinoma growth and metastasis

**Figure 2:**
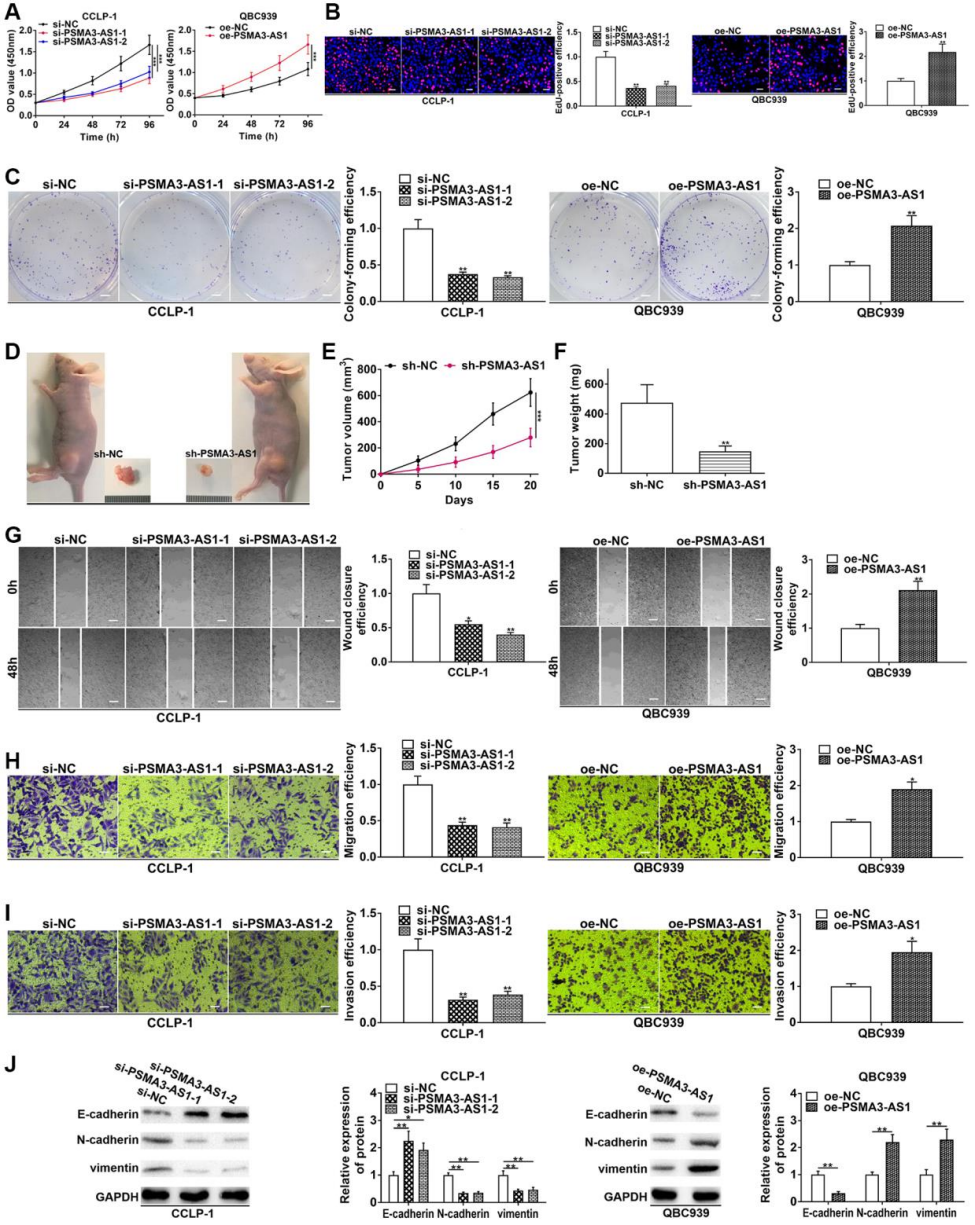
**PSMA3-AS1 promotes CCA cell proliferation, invasion and EMT.** (**A**) CCK-8 and (**B**) EdU assays determined the effect of PSMA3-AS1 on CCA proliferation. (**C**) Colony formation assay determined the effect of PSMA3-AS1 on colony-forming ability of CCA cells. (**D**) The effect of PSMA3-AS1 on CCA proliferation *in vivo*. (**E**) Knocking down PSMA3-AS1 restrained the tumor volume and (**F**) tumor weight in nude mice. (**G**) Wound healing and (**H**, **I**) transwell assays validated the effects of PSMA3-AS1 on migration and invasion of CCA cells. (**J**) The effect of PSMA3-AS1 on EMT process of CCA cells. ^*^P < 0.05, ^**^P < 0.01, ^***^P < 0.001.

In the light of PSMA3-AS1 expression analysis in the four cholangiocarcinoma cells, PSMA3-AS1 level was the highest in CCLP-1 and the lowest in QBC939 cells. Therefore, to obtain a more logical experimental result, we chose to knock down PSMA3-AS1 in CCLP-1 to execute loss-of-function assays, and chose to overexpress it in QBC939 to perform gain-of-function assays. Proliferation experiments confirmed that silencing PSMA3-AS1 depressed cellular vitality, whereas elevating PSMA3-AS1 enhanced cellular vitality (Figure 2A, 2B). Colony formation assay corroborated that PSMA3-AS1 facilitated the colony-forming ability of cholangiocarcinoma cells (Figure 2C). To examine effect of PSMA3-AS1 on CCA proliferation *in vivo*, we established subcutaneous tumor xenograft model (*n* = 6 per group). Silencing PSMA3-AS1 significantly inhibited tumor growth compared with negative control groups (Figure 2D), including the volume (Figure 2E) and weight (Figure 2F) of the tumors. Whereafter, silencing PSMA3-AS1 depressed cholangiocarcinoma migration, whereas elevating PSMA3-AS1 boosted cell migration (Figure 2G, 2H). Transwell invasion assay pre-coated with Matrigel further uncovered that PSMA3-AS1 facilitated CCA cell invasion in comparison with control groups (Figure 2I). Vimentin and N-cadherin represented EMT-related mesenchymal markers, and E-cadherin represented EMT-related epithelial marker. As shown in Figure 2J, knocking down PSMA3-AS1 significantly inhibited vimentin and N-cadherin, but facilitated E-cadherin in CCLP-1. On the contrary, overexpressed PSMA3-AS1 promoted vimentin and N-cadherin expression, whereas depressed E-cadherin in QBC939 cells. Thus, PSMA3-AS1 accelerated the EMT process of CCA. The above functional experiments suggest that PSMA3-AS1 boosts growth and metastasis of cholangiocarcinoma.

### PAX5 transcriptionally activated and enhanced PSMA3-AS1 expression

**Figure 3:**
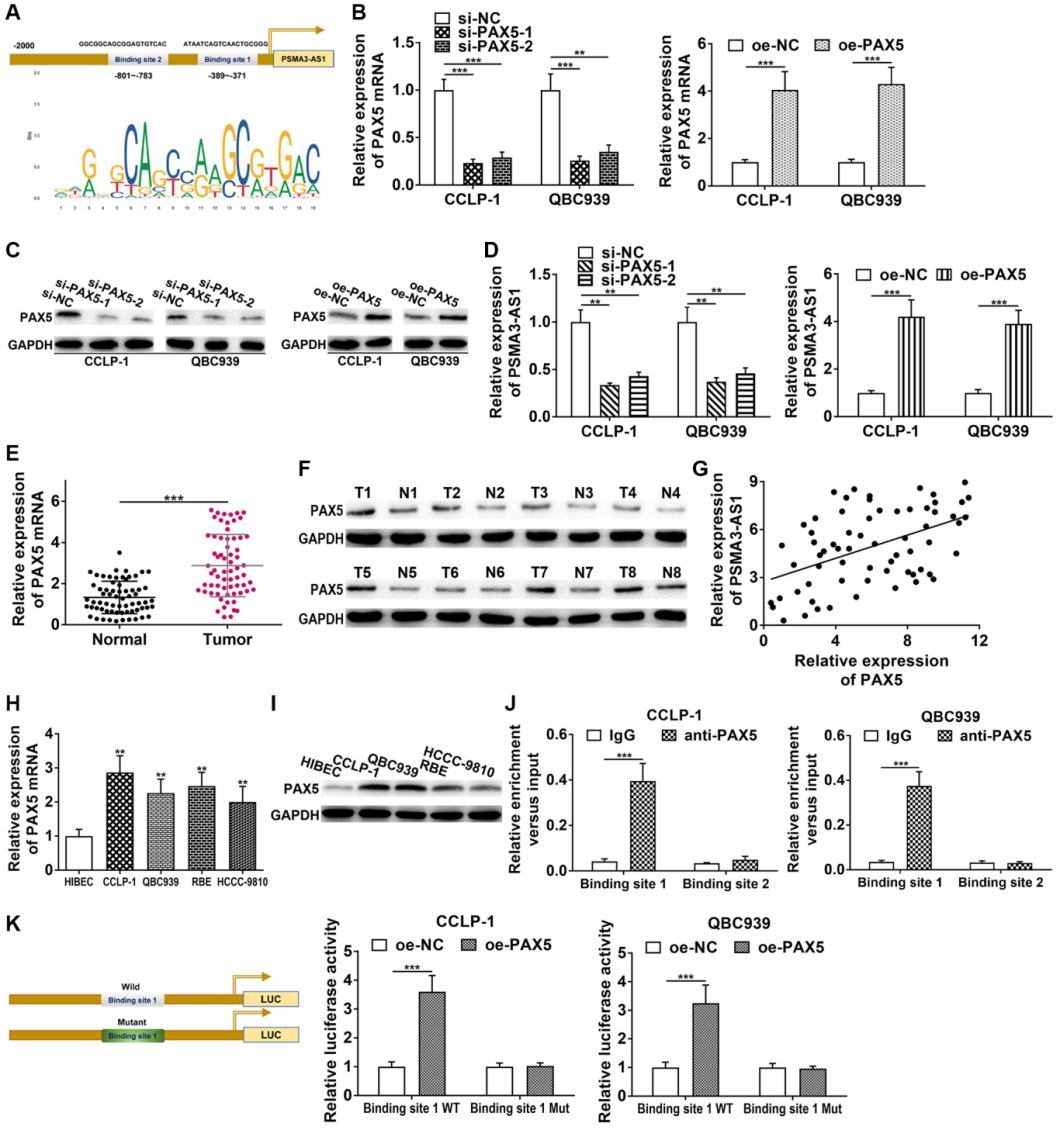
**PAX5 transcriptionally activates and enhances PSMA3-AS1 expression.** (**A**) JASPAR database detected the binding sites of PAX5 and PSMA3-AS1. (**B**) Knockdown efficiency and amplification efficiency of PAX5 in CCA cells both at mRNA and (**C**) protein level. (**D**) PAX5 regulated PSMA3-AS1 expression. (**E**) The expression of PAX5 in CCA tissues both at mRNA and (**F**) protein level. (**G**) PAX5 expression was positively correlated with PSMA3-AS1 expression. (**H**) The expression of PAX5 in CCA cells both at mRNA and (**I**) protein level. (**J**) ChIP assay confirmed that the binding site 1 of PSMA3-AS1 promoter was responsive to PAX5-induced transcription, but no obvious changes in binding site 2. (**K**) Luciferase reporter assay confirmed that the luciferase activity of the wild type of binding site 1 was increased by elevating PAX5, but no significant change in mutant type group. ^**^P < 0.01, ^***^P < 0.001.

We applied JASPAR database (http://jaspar.genereg.net/) to detect the transcriptional regulatory molecules of PSMA3-AS1, and found two PAX5 binding sites in PSMA3-AS1 promoter (Figure 3A). Therefore, we decided to examine their interrelationship. Firstly, we knocked down and overexpressed PAX5 via si-PAX5 and pcDNA3.1-PAX5, and obtained satisfactory knockdown efficiency and amplification efficiency (Figure 3B, 3C). As shown in Figure 3D, knocking down PAX5 significantly inhibited PSMA3-AS1 expression, whereas elevating PAX5 promoted PSMA3-AS1 expression. Next, PAX5 level was increased in CCA tissues both at mRNA and protein level (Figure 3E, 3F). PAX5 expression was positively linked to PSMA3-AS1 (r = 0.4836, *P* < 0.001, Figure 3G). High PAX5 expression in CCA cells was also confirmed (Figure 3H, 3I). In addition, we found that knocking down PAX5 inhibited vimentin and N-cadherin, but facilitated E-cadherin in CCLP-1 (Supplementary Figure 1B); overexpressed PAX5 promoted vimentin and N-cadherin expression, but suppressed E-cadherin in QBC939 (Supplementary Figure 1C). This result illustrated that PAX5 promoted the EMT process of CCA. Chromatin immunoprecipitation (ChIP) confirmed that only binding site 1 responded to PAX5-mediated transcription (Figure 3J). Luciferase reporter experiment further confirmed that elevating PAX5 only strengthened luciferase activity of binding site 1 wild type (Figure 3K). Above findings illustrate that PAX5 expression is upregulated in CCA, and can activate and enhance PSMA3-AS1 transcription.

### PSMA3-AS1 adsorbed and repressed miR-376a-3p in CCA

**Figure 4:**
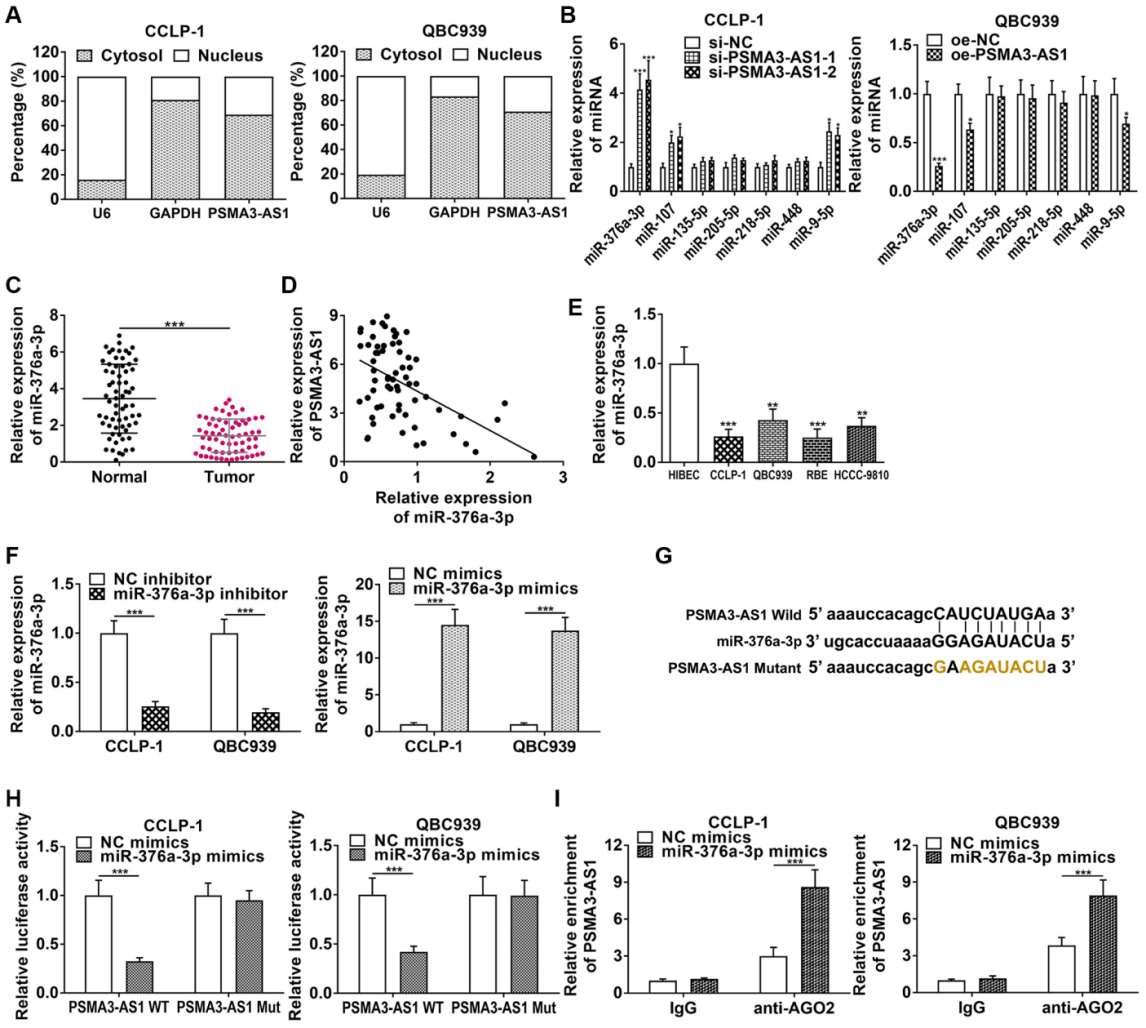
**PSMA3-AS1 competitively binds to miR-376a-3p in CCA cells.** (**A**) Subcellular fractionation assay showed that PSMA3-AS1 was mainly expressed in the cytoplasm of CCA cells. (**B**) PSMA3-AS1 dramatically inhibited miR-376a-3p expression in CCA cells. (**C**) The expression of miR-376a-3p in CCA tissues. (**D**) miR-376a-3p expression was negatively correlated with PSMA3-AS1 expression. (**E**) The expression of miR-376a-3p in CCA cells. (**F**) Knockdown efficiency and amplification efficiency of miR-376a-3p in CCA cells. (**G**, **H**) The luciferase reporter assay indicated that the luciferase activity of PSMA3-AS1 wild type was repressed by co-transfection with miR-376a-3p mimics, but the luciferase activity of PSMA3-AS1 mutant type was not affected. (**I**) AGO2 RIP assay further demonstrated the direct interaction between PSMA3-AS1 and miR-376a-3p. ^*^P < 0.05, ^**^P < 0.01, ^***^P < 0.001.

**Figure 5:**
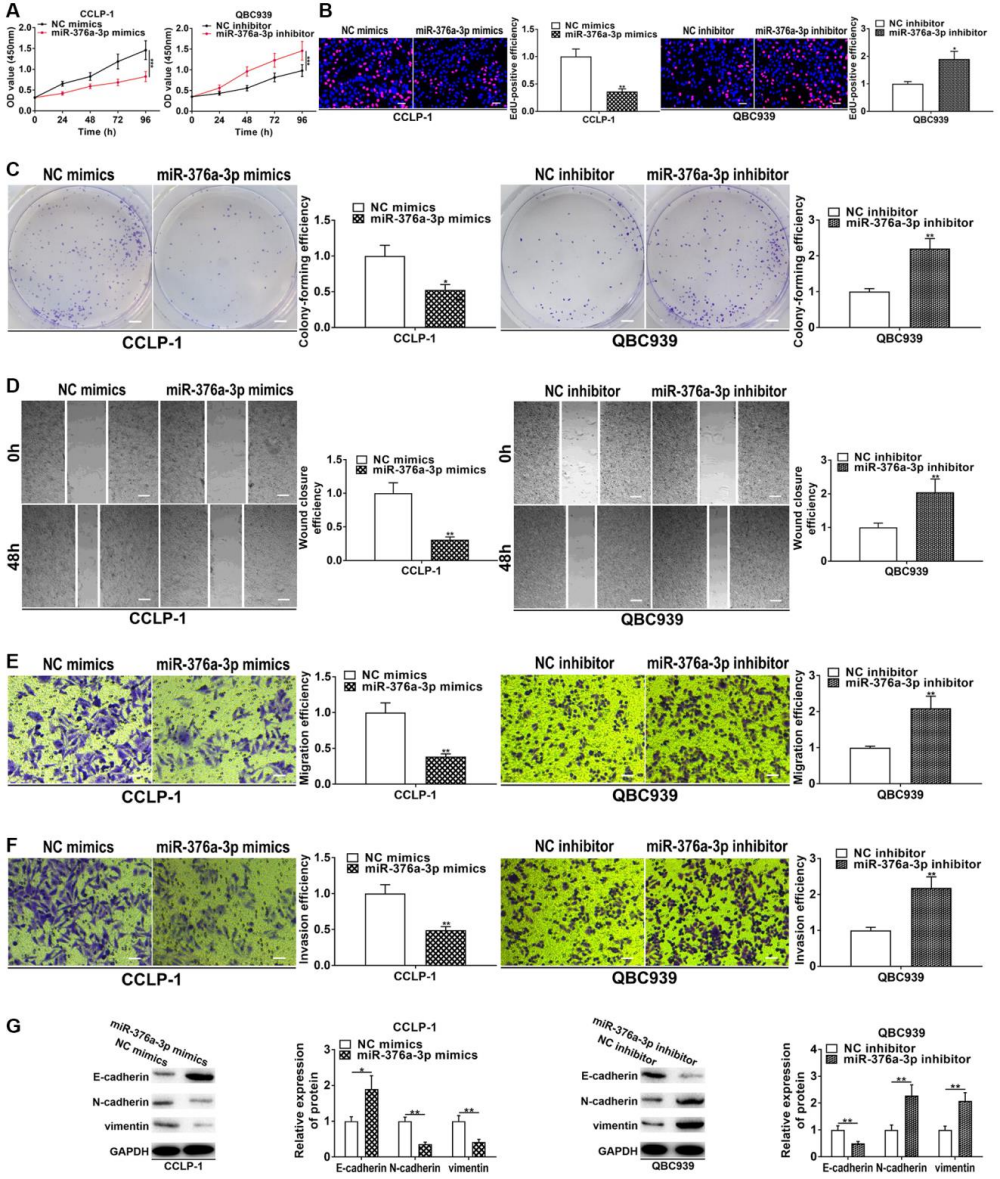
**miR-376a-3p represses CCA cell proliferation, invasion and EMT.** (**A**) CCK-8 and (**B**) EdU assays determined the effect of miR-376a-3p on CCA proliferation. (**C**) Colony formation assay determined the effect of miR-376a-3p on colony-forming ability of CCA cells. (**D**) Wound healing and (**E**, **F**) transwell assays validated the effects of miR-376a-3p on migration and invasion of CCA cells. (**G**) The effect of miR-376a-3p on EMT process of CCA cells. ^*^P < 0.05, ^**^P < 0.01, ^***^P < 0.001.

PSMA3-AS1 was predominantly expressed in CCA cytoplasm (Figure 4A), so we predicted its downstream target genes functioning through ceRNA patterns at the post-transcriptional level via StarBase database. By examining effects of knockdown and overexpression of PSMA3-AS1 on the target miRNAs, we found that knocking down PSMA3-AS1 significantly increased miR-376a-3p level, but overexpressing PSMA3-AS1 significantly decreased miR-376a-3p (Figure 4B). MiR-376a-3p expression was downregulated in CCA tissues (Figure 4C), and negatively related to PSMA3-AS1 expression (r = −0.4969, *P* < 0.001, Figure 4D). MiR-376a-3p was also downregulated in cholangiocarcinoma cells (Figure 4E). The knockdown efficiency and overexpression efficiency of miR-376a-3p inhibitor and mimics were shown in Figure 4F. Elevating miR-376a-3p only repressed luciferase activity of PSMA3-AS1 wild type (Figure 4G, 4H). AGO2 RNA immunoprecipitation (RIP) demonstrated that PSMA3-AS1 directly adsorbed miR-376a-3p (Figure 4I). Besides, overexpressing miR-376a-3p depressed growth and metastasis of cholangiocarcinoma, but knocking down miR-376a-3p promoted these malignant biological behaviors (Figure 5). The above results indicate that PSMA3-AS1 adsorbs miR-376a-3p to accelerate CCA deterioration.

### miR-376a-3p targeted LAMC1 in CCA

**Figure 6:**
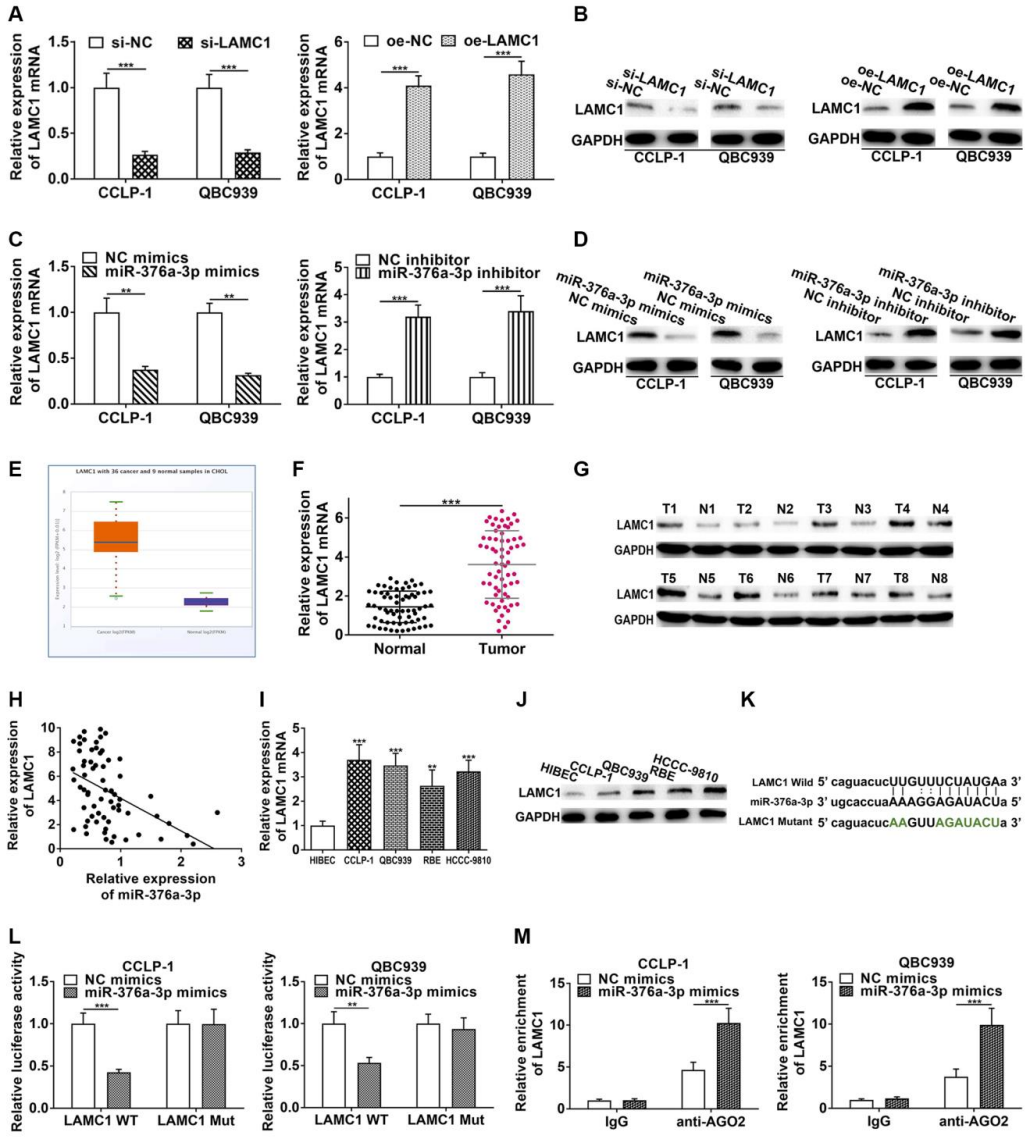
**LAMC1 is directly restrained by miR-376a-3p.** (**A**) Knockdown efficiency and amplification efficiency of LAMC1 in CCA cells both at mRNA and (**B**) protein level. (**C**) miR-376a-3p regulated LAMC1 expression both at mRNA and (**D**) protein level. (**E**) LAMC1 expression in TCGA database. (**F**) The expression of LAMC1 in CCA tissues both at mRNA and (**G**) protein level. (**H**) LAMC1 expression was negatively correlated with miR-376a-3p expression. (**I**) The expression of LAMC1 in CCA cells both at mRNA and (**J**) protein level. (**K**) The wild type and mutant type of LAMC1 3’UTR were cloned into luciferase reporter plasmids, respectively. (**L**) The miR-376a-3p mimics reduced the luciferase activity of LAMC1 wild type rather than mutant type. (**M**) RIP assay uncovered that LAMC1 was enriched by anti-AGO2 antibodies with miR-376a-3p mimics in CCA cells. ^**^P < 0.01, ^***^P < 0.001.

**Figure 7:**
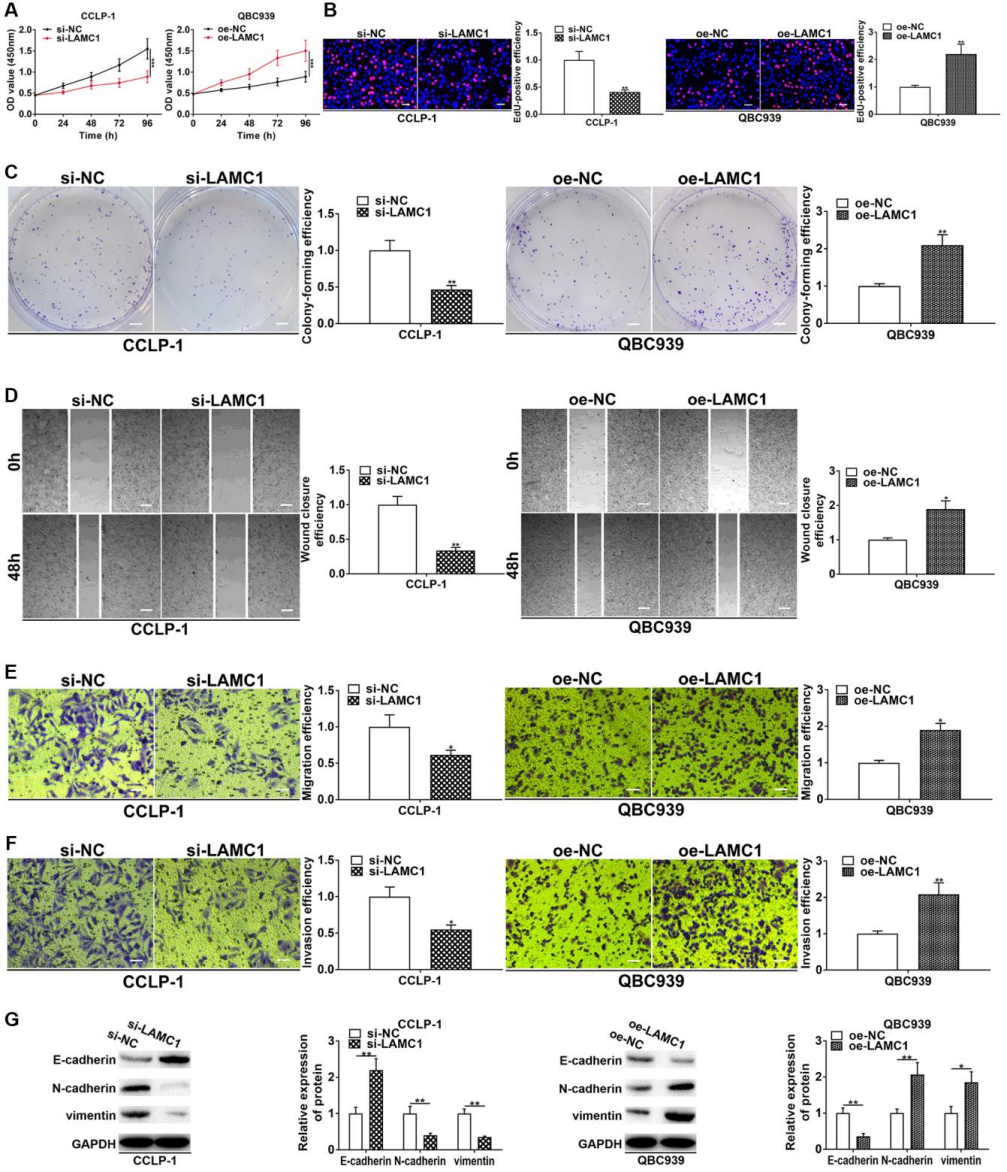
**LAMC1 facilitates CCA cell proliferation, invasion and EMT.** (**A**) CCK-8 and (**B**) EdU assays determined the effect of LAMC1 on CCA proliferation. (**C**) Colony formation assay determined the effect of LAMC1 on colony-forming ability of CCA cells. (**D**) Wound healing and (**E**, **F**) transwell assays validated the effects of LAMC1 on migration and invasion of CCA cells. (**G**) The effect of LAMC1 on EMT process of CCA cells. ^*^P < 0.05, ^**^P < 0.01, ^***^P < 0.001.

StarBase v3.0 database showed that PSMA3-AS1 had overlapping binding sites with LAMC1 at miR-376a-3p sequences (Supplementary Figure 1D). Hence, we determined to validate regulatory relationship of miR-376a-3p and LAMC1. As shown in Figure 6A and 6B, si-LAMC1 significantly inhibited LAMC1 expression, while pcDNA3.1-LAMC1 significantly enhanced LAMC1. Furthermore, miR-376a-3p mimics decreased LAMC1 expression and miR-376a-3p inhibitor increased LAMC1 level in CCA cells (Figure 6C, 6D). Moreover, silencing PAX5 restrained LAMC1 expression and overexpressing PAX5 facilitated LAMC1 expression (Supplementary Figure 1E). TCGA displayed that LAMC1 was upregulated in cholangiocarcinoma (Figure 6E). Authentically, LAMC1 was overexpressed in CCA tissues (Figure 6F, 6G), and negatively related to miR-376a-3p (r = −0.4561, *P* < 0.001, Figure 6H). Nevertheless, LAMC1 was positively related to PAX5 (r = 0.4457, *P* < 0.001, Supplementary Figure 1F). LAMC1 was also elevated in cholangiocarcinoma cells (Figure 6I, 6J). We implemented luciferase reporter assay and RIP assay to demonstrate complementary binding of miR-376a-3p and LAMC1. Wild and mutant type of LAMC1 3′UTR were constructed into luciferase plasmids, respectively (Figure 6K). Elevating miR-376a-3p reduced the luciferase activity of LAMC1 wild type rather than mutant (Figure 6L). Furthermore, RIP uncovered that LAMC1 was augmented via anti-AGO2 antibody with miR-376a-3p mimics (Figure 6M). Above findings illustrate that miR-376a-3p directly binds to LAMC1 and restrains its expression in CCA cells. Besides, we detected influence of LAMC1 on malignant biological behaviors of cholangiocarcinoma, and corroborated that downregulated LAMC1 repressed cellular growth and metastasis, whereas upregulated LAMC1 facilitated these malignant biological behaviors (Figure 7).

### PSMA3-AS1 increased LAMC1 through adsorbing miR-376a-3p to promote CCA progression

**Figure 8:**
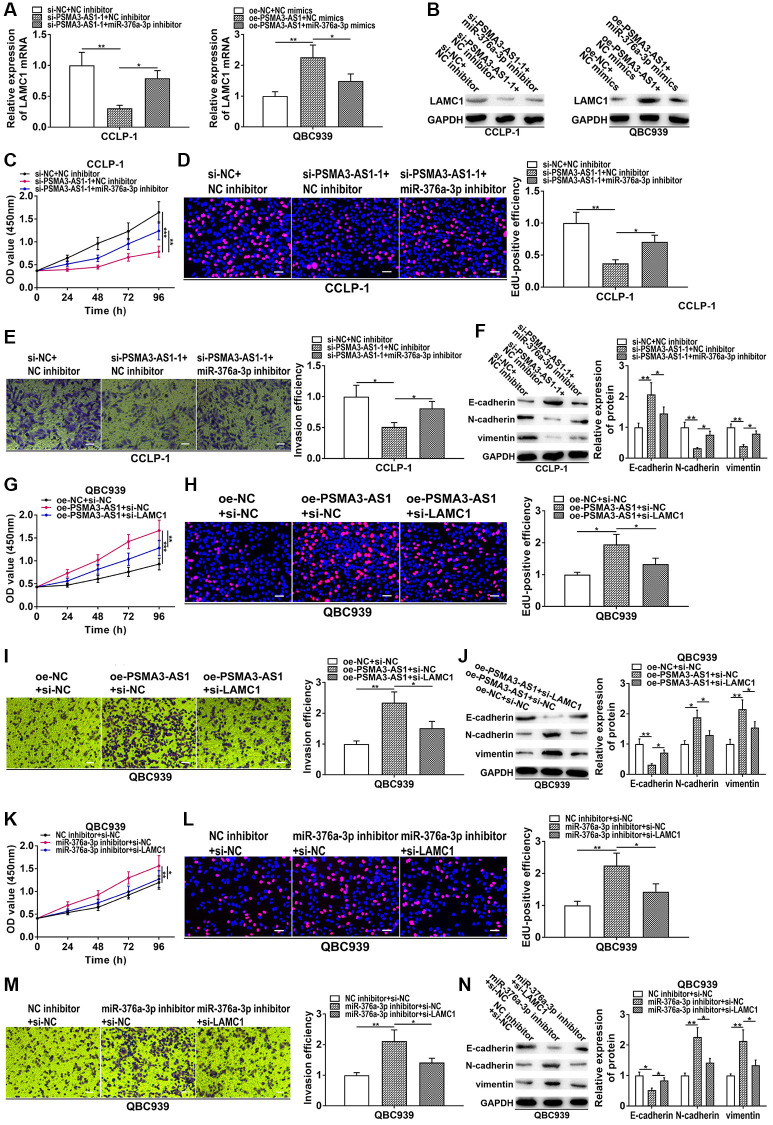
**PSMA3-AS1 up-regulates LAMC1 by sponging miR-376a-3p to accelerate CCA development.** (**A**, **B**) Knockdown of PSMA3-AS1 inhibited LAMC1 expression in CCA cells, but co-transfection of miR-376a-3p inhibitor rescued this process. On the contrary, overexpression of PSMA3-AS1 promoted LAMC1 expression, whereas co-transfection of miR-376a-3p mimics could save the promoting effect caused by PSMA3-AS1 overexpression. (**C**–**F**) Knocking down miR-376a-3p could reverse the inhibition of proliferation, invasion and EMT caused by PSMA3-AS1 knockdown. (**G**–**J**) Downregulated LAMC1 could rescue the promotion of proliferation, invasion and EMT generated by PSMA3-AS1 overexpression. (**K**–**N**) Knocking down miR-376a-3p promoted the proliferation, invasion and EMT process of CCA cells, however, these promotive effects were attenuated by LAMC1 knockdown. ^*^P < 0.05, ^**^P < 0.01, ^***^P < 0.001.

To further confirm that PSMA3-AS1 regulated miR-376a-3p/LAMC1 to boost CCA development, we performed rescue experiments. As presented in Figure 8A and 8B, silencing PSMA3-AS1 inhibited LAMC1 expression in CCA cells, but reducing miR-376a-3p rescued this process. On the contrary, overexpression of PSMA3-AS1 promoted LAMC1 expression, whereas co-transfection of miR-376a-3p mimics could save promoting influence caused by PSMA3-AS1 overexpression. In CCK-8 and EdU experiments, knocking down miR-376a-3p could reverse the proliferative inhibition generated via silencing PSMA3-AS1 (Figure 8C, 8D). In transwell and EMT assays, restoration of miR-376a-3p rescued suppression of metastasis and EMT induced via silencing PSMA3-AS1 (Figure 8E, 8F). Moreover, downregulated LAMC1 could rescue facilitation of growth, metastasis and EMT induced by PSMA3-AS1 overexpression (Figure 8G–8J). In addition, knocking down miR-376a-3p boosted cellular malignant process, nevertheless, the boosting influence was weakened via silencing LAMC1 (Figure 8K–8N). These results suggest that PSMA3-AS1 boosts cholangiocarcinoma proliferation and metastasis by modulating miR-376a-3p/LAMC1.

## DISCUSSION

In recent years, a good deal of researches have revealed that lncRNAs were deregulated in malignant tumors, and became targets for diagnosing and treating cancers, including cholangiocarcinoma [7]. LncRNAs play a role of oncogene or tumor suppressor gene in CCA [18]. Some researches have demonstrated that PSMA3-AS1 exerted cancer-promoting function in some types of tumors [19]. Nevertheless, specific action of PSMA3-AS1 in CCA is unknown. Using the TCGA database, we found abnormally increased PSMA3-AS1 in CCA. It was confirmed via RT-qPCR that PSMA3-AS1 was indeed elevated in cholangiocarcinoma, and significantly linked to worse survival. This is consistent with trends in previous studies. Analysis of PSMA3-AS1 as a prognostic assessment indicator also displayed its satisfactory sensitivity and specificity. Proliferation and invasion are common and important biological behaviors of tumors [20]. In this study, we performed loss-of-function and gain-of-function experiments to confirm that PSMA3-AS1 boosted CCA growth, invasion and EMT process. These suggest that PSMA3-AS1 is not only abnormally highly expressed, but also plays a tumor-promoting role in CCA.

LncRNAs can be involved in gene regulation at epigenetic, transcriptional and post-transcriptional stages through multiple complex molecular mechanisms [8, 21, 22]. PSMA3-AS1 is predominantly expressed in CCA cytoplasm, so we evaluated whether it promotes CCA progression at posttranscriptional level through the ceRNA mechanism. In ceRNA pattern, lncRNAs modulate target genes via competitively binding miRNA [23]. Predicted by the bioinformatic database, we found that PSMA3-AS1 and LAMC1 possessed overlapping binding site of miR-376a-3p. After that, we verified that LAMC1 was enhanced in cholangiocarcinoma and boosted cellular malignant progress. In terms of mechanisms, we confirmed that PSMA3-AS1 upregulated LAMC1 by sponging of miR-376a-3p, thereby expediting cholangiocarcinoma deterioration. Rescue assays further confirmed that PSMA3-AS1 boosted CCA growth and metastasis by regulating miR-376a-3p/LAMC1.

PAX5 is an important cancer-related transcription factor. Previous studies have demonstrated that PAX5 was deregulated in a variety of tumors and involved in malignant biological behaviors such as proliferation and metastasis [14]. By JASPAR database analysis, we found PAX5 binding sites in the PSMA3-AS1 promoter region, and that the relative score was high. According to the literatures, PAX5 as a transcription factor could activate lncRNA transcription, including IDH1-AS1 [15], FOXP4-AS1 [24], LINC01194 [25], UASR1 [26]. Therefore, we attempted to detect the effect of PAX5 on the regulation of PSMA3-AS1 expression. As predicted, we confirmed that the transcription factor PAX5 enhanced PSMA3-AS1 expression by binding to its promoter region. In addition, PAX5 promoted EMT process and LAMC1 expression in CCA. Accordingly, PAX5-induced PSMA3-AS1 boosted occurrence and development of cholangiocarcinoma via mediating miR-376a-3p/LAMC1.

In conclusion, the study reveals that PSMA3-AS1 represents a new cancer-promoting lncRNA in cholangiocarcinoma. PSMA3-AS1 is remarkably increased in CCA and its overexpression presages a terrible prognosis. PAX5-mediated PSMA3-AS1 elevates LAMC1 through adsorbing miR-376a-3p, thereby facilitating cellular growth and metastasis. The present research uncovers that PAX5/PSMA3-AS1/miR-376a-3p/LAMC1 expedites cholangiocarcinoma deterioration, and PSMA3-AS1 is hopeful to become an intervention target for cholangiocarcinoma.

## MATERIALS AND METHODS

### Clinical specimens

66 CCA tissues and matched adjoining non-cancerous bile duct tissues from The 2nd Affiliated Hospital of Harbin Medical University were gathered. The patients had no preoperative radiotherapy and chemotherapy. Collected specimens were promptly placed into liquid nitrogen. Clinical research has been authorized via Ethics Committee of Hospital. Participators signed the informed consent form.

### Cellular cultivation and interference

HCCC-9810, QBC939, RBE, CCLP-1 and normal HIBEC were applied in this study. Cell was incubated in RPMI-1640 and DMEM with 10% FBS (Invitrogen, Carlsbad, CA, USA). SiRNA was applied for knockdown of the target genes, and pcDNA3.1 plasmid was applied for overexpression. They were all purchased from GenePharma (Shanghai, China). Transfection was performed using Lipofectamine 3000 (Invitrogen). SiRNAs were shown in the Supplementary Table 1.

### RT-qPCR

RNAs were extracted via TRIzol (Invitrogen). PrimeScript™ RT Master Mix kit (Takara, Shiga, Japan) was applied for reverse transcription. Finally, RT-qPCR was carried out via SYBR Premix DimerEraser kit (Takara) with ABI Prism 7300 Thermal Cycler (Applied Biosystems, Foster City, CA, USA). GAPDH and U6 were treated as controls. PCR primer sequences were listed in the Supplementary Table 1.

### CCK-8 experiment

Cellular proliferative ability was detected via CCK-8 experiment. 3 × 10^3^ cells were inoculated into 96-well plates. 10 μL CCK-8 reagent (Dojindo, Kumamoto, Japan) was dropped in each well for 2 hours. Absorbance of each well at 450 nm was identified every 24 hours.

### Colony formation experiment

Cell was inoculated in 6-well plate, and then incubated for 2 weeks. The culture was terminated when visible colonies formed. Colony was soaked in formaldehyde and dyed using crystal violet (Beyotime, Beijing, China) for 30 min. Staining diluent was slowly eluted, and colonies were calculated.

### EdU experiment

The EdU kit (Ribobio, Guangzhou, China) was applied to perform EdU incorporation assay. CCA cell was cultivated using 100 μL EdU at 37°C for 2 hours, and dyed successively by Apollo and Hoechst.

### Subcutaneous tumor formation experiment

6-week-old female BALB/c nude mice (Vital River Laboratory Animal Technology Co., Ltd., Beijing, China) were kept in a SPF grade place. Animal research has been authorized via Animal Care and Use Committee of The Second Affiliated Hospital of Harbin Medical University. A total of 5 × 10^6^ CCLP-1 suspension was subcutaneously inoculated into posterior flank of mouse. Xenograft volume was assessed using 0.5 × length × width^2^ per 5 days. Xenograft weight was assessed after 20 days.

### Wound healing assay

For migration assay, CCA cell was inoculated into the 6-well plate. When cells reached 90% confluence, a straight wound was produced using a pipette tip. Then cells were incubated for 48 hours without serum. The wound width was detected at 0 and 48 hours using a microscope, and the migratory ability was evaluated according to the relative wound width.

### Transwell assay

CCA cells in 200 μL serum-free medium were inoculated in superior chamber (Corning Incorporated, Corning, NY, USA) that was inserted in a 24-well plate with 600 μL complete medium in the bottom. After incubation for 24 hours, bottom cell was reacted in formaldehyde and dyed using crystal violet. Invasion experiment was similarly executed in chamber pre-coated with diluted Matrigel (BD Biosciences, San Jose, CA, USA).

### Western blotting

The sample was lysed in RIPA (Beyotime) and the lysates were collected after centrifugation for 15 min at 12000 r/min. Afterwards, protein concentration was measured via BCA (Beyotime). After separation via SDS-PAGE, sample was semi-dry transferred to PVDF membrane (Millipore, Billerica, MA, USA), and then transferred PVDF was soaked using 5% nonfat milk for 2 hours. Subsequently, primary antibody (Abcam, Cambridge, MA) soaked PVDF overnight at 4°C. Secondary antibody (Abcam) soaked PVDF for 1.5 hours.

### ChIP assay

The purpose of ChIP assay (Millipore) is to detect whether the transcription factor PAX5 binds to PSMA3-AS1 promoter region. For produce of DNA-protein cross-links, formaldehyde treatment was conducted on CCA cells. The sonication of cell lysates was used for generating chromatin fragments. Then specific antibodies were used to immunoprecipitate the lysates, and IgG was deemed as control. RT-qPCR was performed to examine precipitated chromatin DNA. The PCR primers were presented in the Supplementary Table 1.

### Luciferase reporter experiment

The purpose of this assay is to examine the interaction of PAX5 with the DNA fragment of the PSMA3-AS1 promoter. This assay is also used to validate binding capacity of miR-376a-3p to PSMA3-AS1/LAMC1. Wild-type (WT) and mutant-type (Mut) plasmids were designed and inserted into pmirGLO reporter vector (Promega, Madison, WI, USA). miR-376a-3p mimics/NC or oe-PAX5/NC was co-transfected with the WT or Mut luciferase plasmids into CCA cell. Luciferase activity was estimated using a dual-luciferase reporter kit (Promega) after 48 hours.

### RIP assay

The aim of AGO2 RIP assay (Millipore) is to affirm interreaction of miR-376a-3p and PSMA3-AS1/LAMC1. Briefly, RIP lysis buffer was applied to lyse CCA cells. Whereafter, RNA was immunoprecipitated via magnetic beads conjugated with anti-AGO2 antibody (Millipore) or IgG. Purified RNA was measured by RT-qPCR.

### Statistical analysis

Data were presented as mean ± SD and analyzed via GraphPad 7.0 and SPSS 20.0. Student’s *t*-test and ANOVA were used to analyze differences. *P* < 0.05 was considered to indicate a statistically significant difference.

## Supplementary Materials

Supplementary Figure 1

Supplementary Table 1
