# Supplementary material for: PSMA3-AS1 induced by transcription factor PAX5 promotes cholangiocarcinoma proliferation, migration and invasion by sponging miR-376a-3p to up-regulate LAMC1
Source: Aging (Albany NY). 2022 Jan 12;14(1):509–25. doi: 10.18632/aging.203828 (PMC8791211; doi:10.18632/aging.203828)
Supplement: Supplementary Figure 1 [file aging-14-203828-s001.pdf]

SUPPLEMENTARY FIGURE

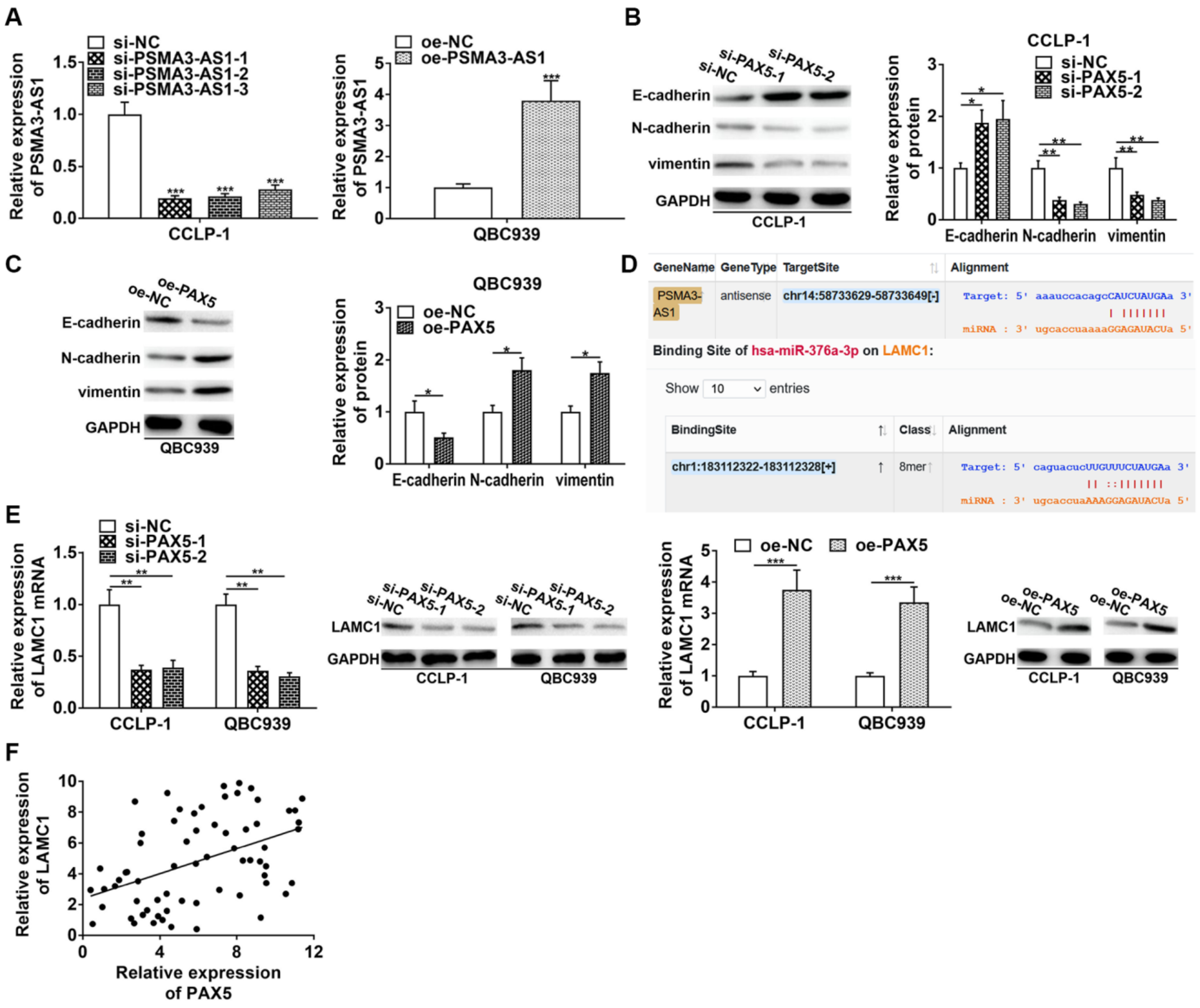

Supplementary Figure 1. PAX5 promotes EMT process and LAMC1 expression in CCA. (**A**) Knockdown efficiency and amplification efficiency of PSMA3-AS1. (**B**) Silencing PAX5 inhibited the EMT process in CCLP-1 cells, and (**C**) overexpressing PAX5 promoted EMT process in QBC939 cells. (**D**) StarBase v3.0 database showed that PSMA3-AS1 had overlapping binding sites with LAMC1 at miR-376a-3p sequences. (**E**) PAX5 promoted LAMC1 expression both at mRNA and protein level in CCA. (**F**) PAX5 expression was positively related to LAMC1 expression in CCA. $^{*}P < 0.05$, $^{**}P < 0.01$, $^{***}P < 0.001$.
