# Supplementary material for: PSMA3-AS1 induced by transcription factor PAX5 promotes cholangiocarcinoma proliferation, migration and invasion by sponging miR-376a-3p to up-regulate LAMC1
Source: Aging (Albany NY). 2022 Jan 12;14(1):509–25. doi: 10.18632/aging.203828 (PMC8791211; doi:10.18632/aging.203828)
Supplement: Supplementary Table 1 [file aging-14-203828-s002.pdf]

SUPPLEMENTARY TABLE

Supplementary Table 1. Primers and siRNAs used in this study.

Names	Sequences (5′ to 3′)
PSMA3-AS1	F: 5′-AACAGACCATCAGAAGAGAACA-3′<br>R: 5′-GAACAGAAACCAGAGCCATACA-3′
LAMC1	F: 5′-ACTCCTAATCTTGGACCATAC-3′<br>R: 5′-ACAACAGCACAACTTGAAC-3′
PAX5	F: 5′-ACTTGCTCATCAAGGTGTCAG-3′<br>R: 5′-TCCTCCAATTACCCCAGGCTT-3′
GAPDH	F: 5′-GACAGTCAGCCGCATCTTCT-3′<br>R: 5′-TTAAAAGCAGCCCTGGTGAC-3′
U6	F: 5′-CTCGCTTCGGCAGCACA-3′<br>R: 5′-AACGCTTCACGAATTTGCGT-3′
miR-376a-3p	F: 5′-TGCACCTAAAAGGAG-3′<br>R: 5′-GTGCAGGGTCCGAGGT-3′
miR-107	F: 5′-GCCGAATTCAAAGCGAGATTCCATCAGCA-3′<br>R: 5′-GCCGGATCCTGTCAACCCAGAACT-CAAAGG-3′
miR-135-5p	F: 5′-AGCATAATACAGCAGGCACAGAC-3′<br>R: 5′-AAAGGTTGTTCTCCACTCTCTCAC-3′
miR-205-5p	F: 5′-TCCTTCATTCCACCGGAGTCTG-3′<br>R: 5′-GCGAGCACAGAATTAATACGAC-3′
miR-218-5p	F: 5′-TTGCGGATGGTTCCGTCAAGCA-3′<br>R: 5′-ATCCAGTGCAGGGTCCGAGG-3′
miR-448	F: 5′-TTATTGCGATGTGTTCCTTATG-3′<br>R: 5′-ATGCATGCCACGGGCATATACACT-3′
miR-9-5p	F: 5′-GTGCAGGGTCCGAGGT-3′<br>R: 5′-GCGCTCTTTGGTTATCTAGC-3′
PSMA3-AS1 promoter binding site 1	F: 5′-GGTAGCGAGGTCCTATCCGA-3′<br>R: 5′-GTATCCCCGGATTCCCCTCT-3′
PSMA3-AS1 promoter binding site 2	F: 5′-GGGAGAGGGATGCTAGGCAA-3′<br>R: 5′-CGCCCGCCTCAACTTTTTG-3′
si-PSMA3-AS1-1	5′-CCAGCAUCAAGAUGAUUUA-3′
si-PSMA3-AS1-2	5′-UCUCGAAAACCCGAAAGAGAA-3′
si-PSMA3-AS1-3	5′-GGCCUAAGAAUCUCUUGAA-3′
si-PAX5-1	5′-GCAGUCUGUUCUGAACAUA-3′
si-PAX5-2	5′-CAAAGGAUAGUGGAACUUG-3′
si-LAMC1	5′-UCAUCAAUCUGAAAGGUAGAG-3′
